# Alcohol Consumption Patterns and Risk of Early-Onset Vasomotor Symptoms in Premenopausal Women

**DOI:** 10.3390/nu14112276

**Published:** 2022-05-29

**Authors:** Ria Kwon, Yoosoo Chang, Yejin Kim, Yoosun Cho, Hye Rin Choi, Ga-Young Lim, Jeonggyu Kang, Kye-Hyun Kim, Hoon Kim, Yun Soo Hong, Jihwan Park, Di Zhao, Sanjay Rampal, Juhee Cho, Eliseo Guallar, Hyun-Young Park, Seungho Ryu

**Affiliations:** 1 Center for Cohort Studies, Total Healthcare Center, Kangbuk Samsung Hospital, Sungkyunkwan University School of Medicine, Seoul 04514, Korea; ria.kwon@samsung.com (R.K.); reenya273@gmail.com (Y.K.); hrchoi7542@gmail.com (H.R.C.); gayoung.lim@samsung.com (G.-Y.L.); jg1980.kang@samsung.com (J.K.); jh1448.cho@samsung.com (J.C.); 2 Institute of Medical Research, School of Medicine, Sungkyunkwan University, Suwon 16419, Korea; 3 Department of Occupational and Environmental Medicine, Kangbuk Samsung Hospital, Sungkyunkwan University School of Medicine, Seoul 04514, Korea; 4 Department of Clinical Research Design & Evaluation, Samsung Advanced Institute for Health Sciences & Technology, Sungkyunkwan University, Seoul 06355, Korea; 5 Total Healthcare Center, Kangbuk Samsung Hospital, Sungkyunkwan University School of Medicine, Seoul 04514, Korea; yoosun.cho@samsung.com; 6 Department of Obstetrics and Gynecology, Kangbuk Samsung Hospital, Sungkyunkwan University School of Medicine, Seoul 03181, Korea; khmd.kim@samsung.com; 7 Department of Obstetrics and Gynecology, Seoul National University College of Medicine, Seoul 03080, Korea; obgyhoon@gmail.com; 8 Departments of Epidemiology and Medicine, and Welch Center for Prevention, Epidemiology, and Clinical Research, Johns Hopkins University Bloomberg School of Public Health, Baltimore, MD 21205, USA; hong.yunsoo@jhu.edu (Y.S.H.); jhpark@jhu.edu (J.P.); dizhao@jhu.edu (D.Z.); eguallar@jhu.edu (E.G.); 9 Department of Social and Preventive Medicine, Centre for Epidemiology and Evidence Based Practice, Faculty of Medicine, Universiti Malaya, Kuala Lumpur 50603, Malaysia; srampal@ummc.edu.my; 10 Department of Precision Medicine, National Institute of Health, Korea Disease Control and Prevention Agency, Cheongju 28159, Korea; mdhypark@gmail.com

**Keywords:** alcohol consumption, hot flashes, night sweats, vasomotor symptoms, premenopausal women

## Abstract

The role of alcohol consumption in the risk of vasomotor symptoms (VMS), the most cardinal climacteric symptoms, is not well established. We examined their relationship with early-onset VMS among premenopausal women. Moderately-to-severely bothersome VMS, the primary outcome, was assessed using the Korean version of the Menopause-Specific Quality of Life questionnaire. The alcohol consumption categories included lifetime abstainer, former drinker, or current drinker, categorized as light, moderate, heavy, and very heavy. Compared with the lifetime-abstinence (reference), the multivariable-adjusted odds ratio (95% CIs) for prevalent VMS in alcohol consumption of <10, 10–19, 20–39, and ≥40 g/day were 1.42 (1.02–1.99), 1.99 (1.27–3.12), 2.06 (1.19–3.57), and 3.52 (1.72–7.20), respectively (*p* trend <0.01). Compared with the lifetime-abstinence, the multivariable-adjusted hazard ratios (95% CIs) for incident bothersome VMS among average alcohol consumption of <10, 10–19, 20–39, and ≥40 g/day were 1.10 (0.85–1.41), 1.03 (0.70–1.51), 1.72 (1.06–2.78), and 2.22 (1.16–4.23), respectively (*p* trend = 0.02). Increased alcohol consumption positively and consistently showed a relationship with increased risk of both prevalent and incident early-onset VMS. Refraining from alcohol consumption may help prevent bothersome VMS in premenopausal women.

## 1. Introduction

Vasomotor symptoms (VMS), the most common (60–80%) cardinal climacteric symptoms in women experiencing menopausal transition, usually peak in the late perimenopausal to early menopausal stages (mean age of 52 to54 years). VMS onset can occur much earlier, even during the premenopausal stage [1,2]. The severity and duration may differ according to the onset of VMS, and early-onset VMS reportedly lasts longer (up to 11.8 years) and is more severe than VMS occurring around the final menstrual period [3]. Moreover, early-onset and long-lasting VMS have been associated with worse psychological profiles and adverse diseases, including subclinical and cardiovascular diseases [4,5]. Previous studies have also reported distinctive features and risk factors of early-onset VMS compared with those of late-onset VMS, although the risk factors for early-onset VMS are not well established [3,5].

Alcohol consumption is a crucial modifiable risk factor for various morbidities [6]. The effects of alcohol consumption on estrogen and other sex hormones are complex [7,8,9,10,11]. Alcohol consumption is associated with increased levels of estrogen [7,8] and androgen [9,10], while long-term moderate alcohol intake is also significantly associated with increased FSH levels and decreased ovarian volume in premenopausal women [11].

Studies addressing the association between alcohol drinking and VMS have reported mixed results [12,13,14,15,16,17], including a positive association [12,13], no association [14,15], and an inverse association [16,17]. However, alcohol consumption in most previous studies was not the main exposure and relied on average alcohol consumption without considering abstainer bias [12,17]. Currently, there are scarce data to address the role of specific drinking patterns in the development of VMS compared with lifetime abstainers as the reference.

Therefore, the present study aimed to investigate (a) the relationship between alcohol consumption patterns and prevalent early-onset VMS in a cross-sectional study and (b) the longitudinal association between alcohol drinking patterns and the development of VMS before menopause in a cohort study of premenopausal Korean women who participated in a regular health check-up program with repeated assessments of VMS using the Menopause-Specific Quality of Life questionnaire.

## 2. Methods

### 2.1. Study Population

We used the data of a cohort study of midlife Korean women recruited from 2014 to 2018 from the Kangbuk Samsung Health Study, a cohort study of Korean health examinees who participated every 1–2 years in comprehensive health check-up examinations previously described [18,19]. Women in the premenopausal stage or early transition stage with an intact uterus were eligible to meet the following criteria: (1) age 42–52 years; (2) no history of hysterectomy, oophorectomy, or hormone replacement therapy; (3) once or more menstrual periods in the three months before enrollment and no amenorrhea for two months or longer; and (4) no history of malignancy, renal failure, and hypo- or hyperthyroidism that can influence menstrual bleed pattern.

**Figure 1:**
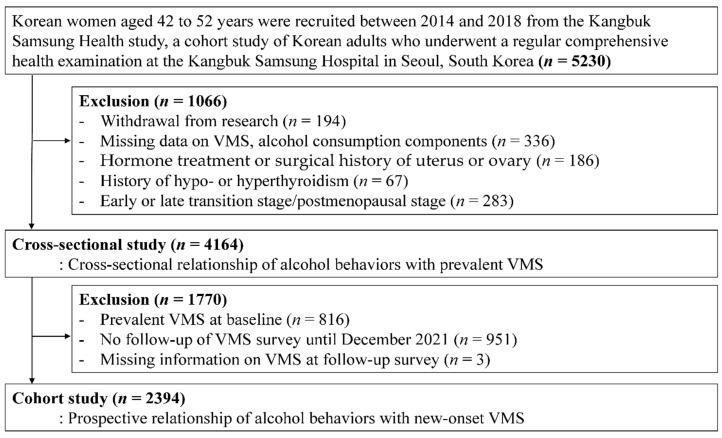
The selection process chart of the participants.

This cohort study was originally designed to evaluate the longitudinal association of menopausal transition with changes in physical and mental health among premenopausal women [20,21]. The menopausal stages were primarily determined based on menstrual bleeding patterns. The present study was restricted to women in the premenopausal stage. Thus, participants with factors that could affect their menstrual cycles were excluded. Among participants who were initially enrolled (*n* = 5230), 1066 women met one or more of the following exclusion criteria (Figure 1): (1) withdrawal; (2) missing information on VMS or alcohol consumption; (3) hormone treatment or surgical history involving the uterus or ovary; (4) history of hypothyroidism or hyperthyroidism, or (5) currently in a later menopausal stage than in the premenopausal stage. Finally, the analytic sample included 4164 premenopausal women in the cross-sectional study. Next, to evaluate the longitudinal association between alcohol consumption and new-onset VMS, women were restricted to those without VMS at baseline who had at least one follow-up VMS assessment before December 2021; therefore, 2396 women were eligible for the cohort study analysis.

The Institutional Review Board of Kangbuk Samsung Hospital (IRB No. KBSMC 2022-04-011) approved this study. All participants provided written informed consent, and all procedures in this study were performed in compliance with the ethical standards of the 1964 Helsinki declaration and its later amendments.

### 2.2. Data Collection

Data on basic characteristics, alcohol drinking behaviors, other lifestyle factors, and history of medical disease or medication was obtained using standardized, self-administered questionnaires at each visit. In contrast, trained nurses and technicians measured blood pressures (BP) and laboratory parameters. Anthropometric measurements, including height and weight, were performed by trained nurses, with participants wearing a lightweight hospital gown with bare feet in a standing position. The widely chosen cutoff value for body mass index (BMI) specific to Asian populations was used to define obesity (≥25 kg/m^2^) [22]. The physical activity categories included inactive, minimally active, and health-enhancing physical activity (HEPA) based on information obtained via the validated Korean version of the International Physical Activity Questionnaire Short Form (IPAQ-SF) [23]. The IPAQ-SF enables the calculation of MET minutes per week (MET level × minutes × number of times per week) for each intensity activity [24]. HEPA participants met any of two criteria: (1) 3 or more days of vigorous activities achieving at least 1500 MET min/week or (2) 7 or more days of any combination of walking and moderate or vigorous activities achieving at least 3000 MET min/week [24]. Other covariates were categorized as never, former, or current smoker for smoking status, and <college graduate or ≥college graduate for education attainment. Hypertension was defined as BP ≥140/90 mmHg, history of physician-diagnosed hypertension, or antihypertensive medication use.

Measurements of fasting serum tests included lipid profiles and liver enzyme (aspartate aminotransferase, alanine transaminase, and gamma-glutamyltransferase [GGT]), high-sensitivity C-reactive protein, glucose, and insulin levels as previously detailed [20,25]. The homeostasis model assessment of insulin resistance was calculated. Diabetes mellitus was defined as glucose-lowering medication use or fasting hyperglycemia based on either serum glucose of ≥126 mg/dL or glycated hemoglobin of ≥6.5%.

### 2.3. Definition of Early-Onset VMS

For VMS assessment at baseline and follow-up, we used the Menopause Specific Quality of Life questionnaire [26]. VMS included hot flashes or night sweats during the past month. If participants had symptoms, they were asked to rate the bothersome degree of each symptom from “Not at all bothered (0 points)” to “Extremely bothered (6 points)”. Participants who answered 0–2 points were classified as having no or mild VMS. On the other hand, participants who answered at the midpoint or higher (3–6 points) were classified as having moderately-to-severely bothersome VMS, which we used as the primary endpoint for the analysis because moderate/severe VMS is likely to negatively influence the quality of life [27,28]. Prevalent VMS was defined as the presence of moderately-to-severely bothersome VMS at baseline, whereas incident VMS was defined as new onset of moderately-to-severely bothersome VMS during follow-up among participants without VMS at baseline.

### 2.4. Definition of Alcohol Consumption

Assessment of alcohol consumption included lifetime abstinence, drinking frequency, and drink amount consumed per drinking day [19]. Drinking status was categorized as lifetime abstainer, former drinker, or current drinker. A lifetime abstainer was defined as someone who never drank alcohol except for ritual sips during inevitable ceremonies. A former drinker was defined as someone who had consumed alcohol in their lifetime, although they were nondrinkers at the time of enrollment. A current drinker’s average daily alcohol consumption was determined by the quantity and frequency of alcohol consumption and classified as light (0.1 to <10), moderate (10 to <20), heavy (20 to <40), or very heavy drinking (≥40 g/day) [19]. Specifically, the questions for the frequency of drinking and number of drinks were “How many days do you drink alcohol in a week on average?” and “How much alcohol do you usually drink per drinking day?”, respectively

For the assessment of binge drinking, we used a specific question from the Alcohol Use Disorders Identification Test for defining binge drinking by the World Health Organization for screening persons with harmful alcohol drinking [29,30]. This question was “How often do you have six or more drinks on one occasion?” with five possible responses (never, less than monthly, monthly, weekly, daily, or almost daily. For the analysis, binge drinking was classified as none, < once a month, once a month, or weekly or more.

### 2.5. Statistical Analysis

The characteristics of the participants are summarized using descriptive statistics according to the alcohol consumption category. As previous studies have reported differences in the risk factors and prognoses among VMS components (hot flashes and night sweats) [31,32,33], we also presented the results for each outcome separately.

For the cross-sectional analysis, the primary dependent variable was the presence of moderately-to-severely bothersome VMS at baseline, including hot flashes and night sweats. Logistic regression models were used to estimate the adjusted odds ratios (OR) and 95% confidence intervals (CIs) for prevalent VMS according to the alcohol consumption category (average alcohol consumption, frequency, and usual quantity consumed) compared with lifetime abstinence (reference). The associations between each VMS component were also evaluated separately.

For the cohort study, the primary endpoint was new-onset moderately-to-severely bothersome VMS occurring before menopause. Each participant was followed from their enrollment to the time of the first reported VMS, the occurrence of menopause, or the end of the study period (31 December 2021), whichever occurred first. When VMS developed between the visit with the first report of VMS and the preceding visit but the exact time of occurrence was unknown, a parametric proportional hazards model was used to account for this type of interval censoring (*stpm* command in STATA) [34] and to calculate the adjusted hazard ratios (HRs) and 95% CIs for new-onset VMS according to the consumption category.

For both cross-sectional and longitudinal relationships between alcohol consumption and VMS risk, the initial model was adjusted for age; multivariable models were further adjusted for education attainment (<12 years, ≥12 years, or missing), physical activity (inactive, minimally active, health-enhancing physical activity, or missing), BMI (continuous), and smoking status (none, former, current smoker, or missing). For the sensitivity analysis, we further adjusted for hypertension (presence, absence, or unknown), and diabetes (presence, absence, or unknown). To evaluate the linear trends in VMS risk, the categories were included as continuous variables in the model.

Furthermore, we evaluated the impact of binge drinking on VMS risk because binge drinking is an independent predictor of adverse health outcomes [29,30,35]. Finally, subgroup analyses were also performed by alcohol flushing (no vs. yes), and their interactions were examined based on likelihood ratio tests while comparing models with and without multiplicative interaction terms.

STATA version 17.0 (STATA Corp LP, College Station, TX, USA) was used for all analyses, with a two-sided statistical significance level of *p* < 0.05.

## 3. Results

### 3.1. Alcohol Consumption and Prevalent Early-Onset VMS

Table 1 shows the characteristics of study participants for the cross-sectional study. The mean age of the 4164 premenopausal women was 44.8 (standard deviation, 2.4) years, and the prevalence of bothersome VMS was 10.2%. Former drinkers tended to be older and had the highest prevalence of obesity. Among current drinkers, those with higher levels of alcohol consumption tended to currently smoke, be obese, physically active, and less educated, and have higher values of BP, high-density lipoprotein cholesterol, triglycerides, and GGT.

Higher alcohol drinking was positively and dose-dependently associated with an elevated prevalence of moderate-to-severe bothersome VMS (Table 2), beginning with the light-drinking category (*p* trend <0.01). After adjusting for confounders, OR (95% CIs) for prevalent VMS comparing light, moderate, heavy, and very heavy drinkers to reference (lifetime abstainer) were 1.42 (1.02–1.99), 1.99 (1.27–3.12), 2.06 (1.19–3.57), and 3.52 (1.72–7.20), respectively (*p* trend <0.01). Current abstainers tended to have a higher prevalence of VMS, especially night sweats; however, this association was not significant. In separate analyses of each symptom (hot flashes and night sweats) as a dependent variable, alcohol drinking patterns were positively related to both symptoms. However, the association with night sweats was stronger than that with hot flashes.

Both higher frequency and quantity showed a dose-response relationship with a higher prevalence of VMS (Table 2). For drinking frequency, the multivariable-adjusted ORs (95% CIs) for overall VMS comparing 1–2 and ≥ 3 days/week to zero days/week as the reference were 1.44 (1.05–1.96) and 2.10 (1.37–3.22), respectively (*p* trend <0.01). For drinking amount per drinking day, the multivariable-adjusted ORs (95% CIs) for VMS comparing 1–2, 3–5, ≥6 to zero drinks/d as the reference category were 1.39 (1.01–1.92), 1.74 (1.21–2.49), and 2.14 (1.34–3.38), respectively (*p* trend <0.01). Similarly, drinking frequency and quantity showed a dose-response association with both hot flashes and night sweat symptoms, although the associations were evident with night sweats. Further adjusting for hypertension and diabetes did not qualitatively change these associations (Table S1).

The frequency of binge drinking also showed an independent and dose-response relationship with an increased prevalence of VMS (*p* trend <0.01) (Table S2). This association was pronounced in analyses of night sweats as a dependent variable, although it was not significant for hot flashes.

### 3.2. Alcohol Consumption and Incidence of Early-Onset VMS

In the longitudinal analysis of premenopausal women without VMS, the average age of the 2394 participants was 44.6 (2.3) years at baseline (Table S3). Similar to the pattern seen in the cross-sectional analysis, former drinkers tended to be older and more obese than current drinkers. Among current drinkers, those with higher levels of alcohol consumption tended to be more hypertensive and less educated and to have higher BP, triglycerides, high-density lipoprotein cholesterol, and GGT. However, the number of participants with high levels of alcohol consumption was small, with only 71 heavy drinkers and 25 very heavy drinkers.

During the follow-up of 11,131.93 person-years (median follow-up, 5.0 years; interquartile range, 3.9–5.9 years; maximum 7.2 years), 546 participants developed new-onset, moderate-to-severe bothersome VMS, and its incidence rate of 4.9 per 100 person-years. Compared with lifetime abstainers, the multivariable-adjusted HRs (95% CIs) for incident VMS were 1.10 (0.85–1.41) for the light drinker, 1.03 (0.70–1.51) for the moderate drinker, 1.72 (1.06–2.78) for the heavy drinker, and 2.22 (1.16–4.23) for very heavy drinker (Table 3). Current abstainers tended to have a higher incidence of VMS with each symptom, although this did not reach statistical significance.

For drinking frequency, compared with zero-day/week as the reference, the multivariable-adjusted HRs (95% CIs) for developing VMS were 1.08 (0.85–1.37) for 1–2 days/week and 1.48 (1.03–2.12) for ≥3 days/week. A significant excess risk was observed for drinking frequency of ≥3 days per week in analyses using night sweats as the outcome but not for using hot flashes as the outcome.

For the drinking amount per drinking day, the increased drinking amount category showed a dose-response and positive association with increased risk of incident VMS and night sweats (*p* trend <0.01), but no significant association with incident hot flashes.

The binge drinking frequency was also positively and dose-dependently associated with an elevated risk of VMS development (Table S1). This pattern was evident for incident night sweats but not for hot flashes.

### 3.3. Effect Modification by Alcohol Flushing Status

The association between alcohol consumption and prevalent VMS differed significantly according to alcohol flushing (*p* for interaction <0.01) (Table S3). Among alcohol nonflushers, the risk of prevalent VMS significantly increased in the heavy drinking category (≥40 g/day), whereas the relative excess risk of prevalent VMS among alcohol flushers was observed beginning in the low-level drinking category (0.1–10 g/d). For the cohort study (Table S4), the effect modification by alcohol flushing status did not statistically differ (*p* interaction = 0.87). In nonflushers, the increased alcohol drinking category showed a positive and dose-response association with a higher risk of developing VMS (*p* trend = 0.02), whereas this pattern was not significant for alcohol flushers (*p* trend = 0.21), possibly owing to the insufficient sample size of moderate and heavy drinking category among alcohol flushers.

## 4. Discussion

In the present cross-sectional and longitudinal studies of premenopausal women, alcohol drinking patterns were dose-dependently associated with an increased risk of bothersome VMS, and the association persisted after adjusting for confounders. In the cross-sectional analysis, the relative excess risk of prevalent VMS was observed from a light drinking level of alcohol consumption measures, including average drinking, drinking frequency, amount consumed per drinking day, and binge drinking. In the cohort analysis, this pattern was similarly observed; however, the incidence of VMS significantly increased among relatively heavy drinkers (≥ 20 g per day). Similarly, higher frequency and quantify of drinking showed dose-dependent association with increased incidence of VMS, with significant excess risk in the highest category of drinking frequency and quantity and the binge drinking frequency. Notably, any levels of alcohol consumption were not protective for prevalent or incident VMS.

Prior research reported conflicting results on the relationship between alcohol consumption and VMS. A study of 1427 middle-aged Finnish women reported that excessive drinking (192 g of pure alcohol per week) showed a positive and cross-sectional association with the prevalent VMS [12]. A cohort study of population-based African American and Caucasian premenopausal women found that a higher number of alcoholic beverages per week was linked to significantly higher odds of hot flashes [13]. Conversely, some previous studies reported opposite or null findings. Another study of 732 women (age range, 45 to 54 years) in the US found that current, moderate, or severe hot flashes were less frequent in women who consumed one drink per day or more compared with nondrinkers [16]. A cohort study of 647 women found that those who drank < 12 drinks or more in the precedent year experienced a shorter duration of hot flashes than that women who drank < 12 drinks in the past year, indicating a potential protective benefit of moderate drinking [17]. A study of 755 peri-menopausal women showed a favorable effect of light alcohol intake (defined as 1 to 5 drinks per week) on the frequency of hot flashes compared with nondrinkers [36].

The reason for conflicting results can be possibly explained by different study populations (e.g., different ethnicity and different menopausal stages) and the fact that alcohol consumption in most of these studies was not the main exposure of interest and was simply classified as a binary category (alcohol use in last year of <12 drinks vs. ≥12 drinks) [17] or as one of three categories (weekly alcohol use of none, 1 to 5 drinks, or ≥6 drinks) [36], thereby limiting the detailed dose-dependent effect and possibly resulting in abstainer bias (former drinkers might have been misclassified as nondrinkers). In contrast to previous findings, we observed no protective effect of alcohol drinking on both prevalent and incident VMS at a low level of alcohol consumption. Instead, our findings suggest that all aspects of average alcohol consumption, frequency, and quantity of usual and binge drinking showed a consistent and positive association with the risk of bothersome VMS in both the cross-sectional and longitudinal studies.

Concerning the individual symptoms of VMS, alcohol consumption showed a stronger association with night sweats compared with hot flashes. This differential association of hot flashes versus night sweats is unclear. A few studies have also reported the differential implications of each or both symptoms, although many studies have mainly focused on hot flashes without considering the different features of each symptom [5,37,38]. Two studies reported associations of both hot flashes and night sweats with the risk of cardiovascular disease [5,37]. Another study reported an association between only night sweats, no hot flashes, with heart disease risk, with plausible explanations that night sweats may reflect the most severe hot flashes or a symptom that is more difficult to tolerate than hot flashes [38]. The mechanisms underlying the differentially stronger relationship between alcohol consumption with night sweats versus hot flashes observed in our study requires further research.

The mechanism underlying the effects of alcohol intake on VMS is not clear yet. Previous research reporting a protective effect of alcohol consumption on VMS [16,17,36] has attributed this effect to the role of alcohol in stimulating estrogen production [39,40]. However, emerging evidence suggests that altered status of other female sex hormones (e.g., increased follicular stimulating hormone [FSH]) or the balance between androgen and estrogen levels, rather than estrogen status alone, may better predict VMS risk [41,42]. Alcohol consumption has been reported to induce both increased androgen levels [9,10] and long-term, moderate alcohol intake may significantly increase FSH levels, a sign of ovarian function decline, in premenopausal women [11]. Therefore, disturbances in reproductive physiology and hormonal dynamics induced by alcohol consumption may contribute to VMS pathogenesis. 

Another possible mechanism involves the role of alcohol in altering hypothalamic temperature regulation [36]. Alcohol can disrupt thermoregulation not only by acting on peripheral blood vessels with vasodilatory properties but also by exerting direct action on the central nervous system [43]. Reportedly, the core temperature may be altered following alcohol intake, which can potentially cause a temporary shift in the sweating threshold [43]. The resulting changes in the temperature setpoint may lead to a series of compensatory mechanisms to dissipate or preserve heat [44], which is thought to be a key driver of VMS pathogenesis [45]. 

Alcohol can also trigger changes in the metabolism of neurotransmitters, such as serotonin (5-hydroxytryptamine [5-HT]) or norepinephrine, which also play a crucial role in temperature regulation and VMS pathophysiology [46]. Alcohol consumption stimulates the release of 5-HT and acts as a stressor that stimulates the upregulation of certain types of 5-HT receptors [47]. The upregulation of 5-HT receptors may disturb the thermoregulatory system in the hypothalamus, causing hot flashes [47]. However, these putative mechanisms are largely speculative; more extensive mechanistic research is required to understand the association of alcohol consumption with increased VMS risk.

Our study had several limitations. First, we relied on a self-reported questionnaire to evaluate the presence and degree of VMS based on subjective symptoms and not objective tests for VMS measurement. Although there was a misclassification risk, the effects of nondifferential misclassification would only induce bias towards the null. Second, alcohol consumption assessment was also determined using a self-administered, structured questionnaire; thus, drinking pattern tends to be under-reported, although the assessment of alcohol consumption assessment relies on self-reporting in most epidemiological studies [48]. 

In addition, we did not consider the differential effect of different alcoholic beverages. Therefore, the possibility of measurement errors and unmeasured confounders cannot be excluded. Third, questions regarding dietary assessment were not mandatory, and information on diet was available for only approximately half of the participants [20]. In addition, at baseline, health examinations and alcohol consumption and VMS assessments were performed on the same day, although follow-up surveys for VMS assessments were not conducted during health examinations on the same day. Thus, we could not incorporate diet information and the changing status of covariates as time-varying covariates in the analysis. Finally, our study population was recruited from health screening centers, mostly covering employees and their spouses with relatively high education levels and no comorbidities. The present study findings are required to be confirmed in other populations with different ethnicities or characteristics.

However, our study had some strengths, including a large-scale study of middle-aged women, the use of prevalent and incident VMS occurring prior to menopause as the outcome, and minimization of abstainer bias by differentiating former drinkers from nondrinkers along with relatively detailed drinking patterns, which enabled us to demonstrate the independent and dose-dependent association of alcohol drinking patterns with risk of both prevalent and incident VMS.

## 5. Conclusions

In the cohort of middle-aged Korean premenopausal stage women, the VMS risk significantly increased with increased alcohol consumption, beginning in the light-drinking category. A positive association between alcohol consumption patterns and VMS risk was consistently found concerning drinking frequency, quantity per episode, and binge drinking frequency. Our findings suggest that refraining from alcohol consumption may help decrease bothersome VMS among women experiencing the menopausal transition, even in the premenopausal stage.

## Figures and Tables

**Table 1:** Characteristics of premenopausal women in the cross-sectional study by drinking category (*n* = 4164).

Characteristics	Total	Drinking Status					
		Lifetime Abstainer	Former Drinker	0 to <10 g/day	10 to <20 g/day	20 to <40 g/day	≥40 g/day
Number (%)	4164	569 (13.7)	172 (4.1)	2879 (69.1)	328 (7.9)	160 (3.8)	56 (1.3)
Age (years) *	44.8 ± 2.4	45.6 ± 2.6	45.3 ± 2.8	44.6 ± 2.3	44.6 ± 2.4	44.8 ± 2.5	44.7 ± 2.4
Age at menarche (years) *	13.9 ± 1.4	14.0 ± 1.4	13.9 ± 1.5	13.9 ± 1.4	14.0 ± 1.5	14.1 ± 1.6	14.0 ± 1.5
Obesity (%) ^a^	16.2	16.3	20.9	15.7	16.5	18.8	19.6
High physical activity (%) ^b^	14.8	13.5	17.4	14.1	18.3	19.4	21.4
Current smoker (%)	1.9	0.7	0.6	1.2	3.7	11.4	17.9
High education (%) ^c^	80.8	80.9	78.4	82.8	73.7	66.9	66.1
Hypertension (%) ^d^	4.5	5.3	3.5	3.7	6.7	11.3	10.7
Systolic BP (mmHg) *	103.9 ± 11.7	104.2 ± 11.8	104.1 ± 12.0	103.2 ± 11.4	105.5 ± 11.4	109.0 ± 13.9	109.8 ± 13.3
Diastolic BP (mmHg) *	66.8 ± 9.2	66.3 ± 8.8	65.5 ± 9.6	66.5 ± 8.9	68.5 ± 9.7	71.6 ± 11.0	72.4 ± 11.3
Diabetes (%) ^e^	1.7	1.2	0.6	1.6	2.1	3.8	1.8
Glucose (mg/dL) *	93.0 ± 12.0	92.4 ± 10.4	94.0 ± 20.9	92.7 ± 11.7	94.0 ± 8.8	96.1 ± 15.7	93.2 ± 15.7
LDLC (mg/dL) *	119.2 ± 28.7	123.5 ± 29.0	120.2 ± 27.7	118.7 ± 28.1	119.8 ± 30.6	115.5 ± 33.0	108.2 ± 30.5
HDLC (mg/dL) *	67.0 ± 15.9	65.0 ± 15.4	66.0 ± 15.1	66.6 ± 15.6	69.3 ± 16.3	74.1 ± 18.8	77.0 ± 20.4
Triglycerides (mg/dL) ^†^	74.0 (57.0–100.0)	77.0 (58.0–105.0)	73.5 (56.0–100.0)	73.0 (57.0–98.0)	78.5 (57.0–106.0)	78.5 (62.0–105.0)	77.0 (63.5–109.5)
AST (U/l) ^†^	18.0 (15.0–20.0)	18.0 (15.0–20.0)	18.0 (16.0–21.0)	17.0 (15.0–20.0)	18.0 (15.0–21.0)	18.0 (16.0–21.0)	18.0 (16.0–20.0)
ALT (U/l) ^†^	13.0 (11.0–17.0)	14.0 (11.0–17.0)	14.5 (12.0–19.0)	13.0 (11.0–17.0)	13.0 (11.0–17.0)	13.0 (11.0–17.0)	13.0 (10.5–15.5)
GGT (U/l) ^†^	13.0 (11.0–18.0)	13.0 (10.0–17.0)	13.0 (11.0–16.0)	13.0 (11.0–18.0)	15.0 (12.0–21.0)	17.0 (13.0–21.0)	18.0 (14.0–30.0)
HOMA-IR ^†^	1.1 (0.7–1.6)	1.1 (0.7–1.6)	1.1 (0.8–1.9)	1.1 (0.7–1.6)	1.2 (0.8–1.6)	1.0 (0.7–1.4)	0.9 (0.6–1.2)
hsCRP (mg/L) ^†^	0.03 (0.02–0.06)	0.03 (0.02–0.06)	0.03 (0.02–0.07)	0.03 (0.02–0.06)	0.03 (0.02–0.05)	0.04 (0.02–0.06)	0.03 (0.02–0.05)

Abbreviations: ALT, alanine aminotransferase; AST, aspartate aminotransferase; BP, blood pressure; GGT, gamma-glutamyl transpeptidase; HDL-C, high-density lipoprotein cholesterol; HOMA-IR, homeostasis model assessment of insulin resistance; hsCRP, high-sensitivity C-reactive protein; LDLC, low-density lipoprotein cholesterol; MET, metabolic equivalents. Data are presented as * means ± standard deviations, ^†^ medians (interquartile ranges), or percentages. ^a^ body mass index ≥25 kg/m^2^; ^b^ defined as either ≥3 days of vigorous activities achieving at least 1500 MET min/week, or ≥7 days of any combination of walking and moderate or vigorous activity achieving at least 3000 MET min/week; ^c^ ≥college graduate; ^d^ defined as either blood pressure ≥140/90 mmHg, history of physician-diagnosed hypertension, or antihypertensive medication use; ^e^ glucose-lowering medication use, fasting hyperglycemia based on either serum glucose of ≥126 mg/dL or glycated hemoglobin of ≥6.5%.

**Table 2:** Association between alcohol consumption and prevalence of early-onset, moderately-to-severely bothersome VMS (overall and each component including hot flash or night sweat) among premenopausal women (*n* = 4164).

Alcohol Consumption Patterns	Vasomotor Symptoms		Hot Flash Symptoms		Night Sweat Symptoms	
	Age-Adjusted OR (95% CI)	Multivariable-Adjusted OR * (95% CI)	Age-Adjusted OR (95% CI)	Multivariable-Adjusted OR * (95% CI)	Age-Adjusted OR (95% CI)	Multivariable-Adjusted OR * (95% CI)
Drinking status						
Lifetime abstainer	Reference	Reference	Reference	Reference	Reference	Reference
Current drinker						
0.1 to <10 g/day	1.41 (1.01–1.97)	1.42 (1.02–1.99)	1.30 (0.91–1.84)	1.31 (0.92–1.86)	1.48 (0.85–2.58)	1.50 (0.86–2.63)
10 to <20 g/day	2.05 (1.32–3.19)	1.99 (1.27–3.12)	1.83 (1.14–2.92)	1.73 (1.07–2.78)	2.82 (1.43–5.58)	2.82 (1.42–5.63)
20 to <40 g/day	2.24 (1.32–3.83)	2.06 (1.19–3.57)	1.33 (0.70–2.51)	1.17 (0.61–2.25)	4.82 (2.34–9.93)	4.51 (2.15–9.48)
≥40 g/day	3.89 (1.94–7.80)	3.52 (1.72–7.20)	2.32 (1.03–5.25)	2.04 (0.88–4.72)	8.98 (3.80–21.26)	8.30 (3.40–20.28)
*p* trend	<0.01	<0.01	0.01	0.06	<0.01	<0.01
Former drinker	1.08 (0.58–2.03)	1.06 (0.56–1.99)	1.10 (0.57–2.11)	1.06 (0.55–2.05)	1.62 (0.65–4.04)	1.59 (0.63–3.99)
Frequency of drinking (days/week)						
0	Reference	Reference	Reference	Reference	Reference	Reference
1–2	1.43 (1.05–1.95)	1.44 (1.05–1.96)	1.31 (0.95–1.81)	1.31 (0.95–1.82)	1.54 (0.94–2.52)	1.54 (0.94–2.53)
≥3	2.15 (1.41–3.27)	2.10 (1.37–3.22)	1.63 (1.03–2.59)	1.59 (0.99–2.54)	3.23 (1.75–5.96)	3.08 (1.64–5.75)
*p* trend	<0.01	<0.01	0.03	0.04	<0.01	<0.01
Number of drinks drinking day						
0	Reference	Reference	Reference	Reference	Reference	Reference
1–2	1.37 (0.99–1.89)	1.39 (1.01–1.92)	1.28 (0.91–1.80)	1.31 (0.93–1.84)	1.29 (0.77–2.15)	1.29 (0.77–2.17)
3–5	1.77 (1.24–2.52)	1.74 (1.21–2.49)	1.57 (1.08–2.28)	1.53 (1.05–2.23)	2.14 (1.24–3.68)	2.11 (1.22–5.65)
≥6	2.40 (1.54–3.76)	2.14 (1.34–3.38)	1.75 (1.06–2.87)	1.53 (0.92–2.54)	3.50 (1.85–6.62)	3.01 (1.56–5.80)
*p* trend	<0.01	<0.01	<0.01	0.03	<0.01	<0.01

Abbreviations: BMI, body mass index; CI, confidence interval; OR, odds ratio; VMS, vasomotor symptoms. Logistic regression model was used to calculate the odds ratio and 95% confidence intervals for moderate-to-severe VMS. * The multivariable model was adjusted for age, attainment, smoking, physical activity level, and BMI.

**Table 3:** Longitudinal association between alcohol consumption and incidence of early-onset, moderately-to-severely bothersome VMS (overall and each component including hot flash or night sweat) among premenopausal women (*n* = 2394).

Alcohol Drinking Patterns	Vasomotor Symptoms		Hot Flash Symptoms		Night Sweat Symptoms	
	Age-Adjusted HRs (95% CI)	Multivariable-Adjusted HR (95% CI) *	Age-Adjusted HRs (95% CI)	Multivariable-Adjusted HR (95% CI) *	Age-Adjusted HRs (95% CI)	Multivariable-Adjusted HR (95% CI) *
Drinking status						
Lifetime abstainer	Reference	Reference	Reference	Reference	Reference	Reference
Current drinker						
0.1 to <10 g/day	1.09 (0.85–1.41)	1.10 (0.85–1.41)	0.99 (0.74–1.34)	1.00 (0.75–1.35)	1.21 (0.89–1.64)	1.21 (0.89–1.64)
10 to <20 g/day	1.09 (0.74–1.59)	1.03 (0.70–1.51)	0.86 (0.53–1.39)	0.82 (0.51–1.35)	1.19 (0.75–1.88)	1.12 (0.71–1.77)
20 to <40 g/day	1.74 (1.09–2.79)	1.72 (1.06–2.78)	1.34 (0.74–2.41)	1.28 (0.70–2.34)	2.05 (1.19–3.52)	1.95 (1.12–3.40)
≥40 g/day	2.23 (1.18–4.24)	2.22 (1.16–4.23)	1.30 (0.52–3.26)	1.32 (0.52–3.32)	3.20 (1.61–6.34)	3.12 (1.56–6.23)
*p* trend	<0.01	0.02	0.59	0.70	<0.01	<0.01
Former drinker	1.29 (0.80–2.05)	1.27 (0.79–2.04)	1.09 (0.62–1.94)	1.08 (0.61–1.91)	1.17 (0.65–2.12)	1.15 (0.63–2.09)
Frequency of drinking (days/week)						
0	Reference	Reference	Reference	Reference	Reference	Reference
1–2	1.09 (0.86–1.38)	1.08 (0.85–1.37)	0.99 (0.74–1.31)	1.00 (0.75–1.32)	1.19 (0.89–1.59)	1.18 (0.89–1.57)
≥3	1.50 (1.05–2.13)	1.48 (1.03–2.12)	1.30 (0.84–1.99)	1.30 (0.84–2.02)	1.65 (1.09–2.51)	1.61 (1.06–2.46)
*p* trend	0.05	0.06	0.39	0.37	0.03	0.04
Number of drinks a drinking day						
0	Reference	Reference	Reference	Reference	Reference	Reference
1–2	1.04 (0.82–1.33)	1.04 (0.81–1.33)	0.95 (0.72–1.27)	0.96 (0.72–1.28)	1.15 (0.86–1.55)	1.14 (0.85–1.54)
3–5	1.21 (0.91–1.61)	1.20 (0.90–1.59)	0.98 (0.70–1.40)	0.98 (0.70–1.38)	1.43 (1.02–2.00)	1.41 (1.00–1.97)
≥6	1.55 (1.05–2.28)	1.47 (1.00–2.17)	1.37 (0.86–2.17)	1.31 (0.82–2.09)	1.79 (1.15–2.80)	1.66 (1.05–2.62)
*p* trend	0.01	0.03	0.34	0.45	<0.01	<0.01

Abbreviations: BMI, body mass index; CI, confidence interval; HR, hazard ratio; VMS, vasomotor symptoms. Parametric proportional hazard models were used to estimate hazard ratios (HRs) and 95% CIs for incident VMS. * The multivariable model was adjusted for age, attainment, smoking, physical activity level, and BMI.

## Data Availability

The data presented in this study are available on request from the corresponding author. The data are not publicly available due to ethical requirements.

## Supplementary Materials

The following supporting information can be downloaded at: https://www.mdpi.com/article/10.3390/nu14112276/s1, Table S1: Cross-sectional and longitudinal association between alcohol consumption and risk of early-onset, moderately to severely bothersome VMS (overall and each components including hot flash or night sweat) among premenopausal women after further adjustment for hypertension and diabetes; Table S2: Prevalence and incidence of early-onset, moderately to severely bothersome VMS among premenopausal women by frequency of binge drinking; Table S3: Cohort study characteristics of premenopausal women without vasomotor symptoms at baseline by drinking category (*n* = 2394); Table S4: Longitudinal association between alcohol consumption and incidence of early-onset bothersome vasomotor symptoms among premenopausal women by alcohol flushing.

## References

[1] Thurston R.C., Joffe H. (2011). Vasomotor symptoms and menopause: Findings from the Study of Women’s Health across the Nation. Obstet. Gynecol. Clin..

[2] Kronenberg F. (1990). Hot flashes: Epidemiology and physiology. Ann. N. Y. Acad. Sci..

[3] Avis N.E., Crawford S.L., Greendale G., Bromberger J.T., Everson-Rose S.A., Gold E.B., Hess R., Joffe H., Kravitz H.M., Tepper P.G. (2015). Duration of Menopausal Vasomotor Symptoms Over the Menopause Transition. JAMA Intern. Med..

[4] Thurston R.C., El Khoudary S.R., Tepper P.G., Jackson E.A., Joffe H., Chen H.-Y., Matthews K.A., Harlow S., Sowers M. (2016). Trajectories of vasomotor symptoms and carotid intima media thickness in the Study of Women’s Health Across the Nation. Stroke.

[5] Zhu D., Chung H.F., Dobson A.J., Pandeya N., Anderson D.J., Kuh D., Hardy R., Brunner E.J., Avis N.E., Gold E.B. (2020). Vasomotor menopausal symptoms and risk of cardiovascular disease: A pooled analysis of six prospective studies. Am. J. Obstet. Gynecol..

[6] Griswold M.G., Fullman N., Hawley C., Arian N., Zimsen S.R., Tymeson H.D., Venkateswaran V., Tapp A.D., Forouzanfar M.H., Salama J.S. (2018). Alcohol use and burden for 195 countries and territories, 1990–2016: A systematic analysis for the Global Burden of Disease Study 2016. Lancet.

[7] Erol A., Ho A.M.C., Winham S.J., Karpyak V.M. (2019). Sex hormones in alcohol consumption: A systematic review of evidence. Addict. Biol..

[8] Mendelson J.H., Lukas S.E., Mello N.K., Amass L., Ellingboe J., Skupny A. (1988). Acute alcohol effects on plasma estradiol levels in women. Psychopharmacology.

[9] Sarkola T., Fukunaga T., Mäkisalo H., Peter Eriksson C. (2000). Acute effect of alcohol on androgens in premenopausal women. Alcohol Alcohol..

[10] Frias J., Torres J., Miranda M., Ruiz E., Ortega E. (2002). Effects of acute alcohol intoxication on pituitary–gonadal axis hormones, pituitary–adrenal axis hormones, β-endorphin and prolactin in human adults of both sexes. Alcohol Alcohol..

[11] Li N., Fu S., Zhu F., Deng X., Shi X. (2013). Alcohol intake induces diminished ovarian reserve in childbearing age women. J. Obstet. Gynaecol. Res..

[12] Moilanen J., Aalto A.M., Hemminki E., Aro A.R., Raitanen J., Luoto R. (2010). Prevalence of menopause symptoms and their association with lifestyle among Finnish middle-aged women. Maturitas.

[13] Freeman E.W., Sammel M.D., Grisso J.A., Battistini M., Garcia-Espagna B., Hollander L. (2001). Hot flashes in the late reproductive years: Risk factors for African American and Caucasian women. J. Women’s Health Gend. Based Med..

[14] Schwingl P.J., Hulka B.S., Harlow S.D. (1994). Risk factors for menopausal hot flashes. Obstet. Gynecol..

[15] Gold E.B., Block G., Crawford S., Lachance L., FitzGerald G., Miracle H., Sherman S. (2004). Lifestyle and demographic factors in relation to vasomotor symptoms: Baseline results from the Study of Women’s Health Across the Nation. Am. J. Epidemiol..

[16] Gallicchio L., Miller S.R., Kiefer J., Greene T., Zacur H.A., Flaws J.A. (2015). Risk factors for hot flashes among women undergoing the menopausal transition: Baseline results from the Midlife Women’s Health Study. Menopause.

[17] Smith R.L., Gallicchio L., Miller S.R., Zacur H.A., Flaws J.A. (2016). Risk factors for extended duration and timing of peak severity of hot flashes. PLoS ONE.

[18] Chang Y., Ryu S., Sung K.-C., Cho Y.K., Sung E., Kim H.-N., Jung H.-S., Yun K.E., Ahn J., Shin H. (2019). Alcoholic and non-alcoholic fatty liver disease and associations with coronary artery calcification: Evidence from the Kangbuk Samsung Health Study. Gut.

[19] Han S.Y., Chang Y., Kim Y., Choi C.Y., Ryu S. (2022). A Dose–Response Relationship of Alcohol Consumption with Risk of Visual Impairment in Korean Adults: The Kangbuk Samsung Health Study. Nutrients.

[20] Choi H.R., Chang Y., Kim Y., Cho Y., Kang J., Kwon M.J., Kwon R., Lim G., Kim K.H., Kim H. (2022). Ideal cardiovascular health metrics and risk of incident early-onset vasomotor symptoms among premenopausal women. J. Clin. Endocrinol. Metab..

[21] Namgoung S., Chang Y., Woo C.Y., Kim Y., Kang J., Kwon R., Lim G., Choi H.R., Kim K.H., Kim H. (2022). Metabolically healthy and unhealthy obesity and risk of vasomotor symptoms in premenopausal women: Cross-sectional and cohort studies. BJOG Int. J. Obstet. Gynaecol..

[22] World Health Organization (2000). The Asia-Pacific Perspective: Redefining Obesity and Its Treatment.

[23] Craig C.L., Marshall A.L., Sjöström M., Bauman A.E., Booth M.L., Ainsworth B.E., Pratt M., Ekelund U., Yngve A., Sallis J.F. (2003). International physical activity questionnaire: 12-country reliability and validity. Med. Sci. Sports Exerc..

[24] Forde C. (2018). Scoring the International Physical Activity Questionnaire (IPAQ).

[25] Cho I.Y., Chang Y., Kang J.H., Kim Y., Sung E., Shin H., Wild S.H., Byrne C.D., Ryu S. (2022). Long or Irregular Menstrual Cycles and Risk of Prevalent and Incident Nonalcoholic Fatty Liver Disease. J. Clin. Endocrinol. Metab..

[26] Park J.-H., Bae S.H., Jung Y.-M. (2020). Validity and Reliability of the Korean Version of the Menopause-Specific Quality of Life. J. Korean Acad. Nurs..

[27] Nappi R.E., Kroll R., Siddiqui E., Stoykova B., Rea C., Gemmen E., Schultz N.M. (2021). Global cross-sectional survey of women with vasomotor symptoms associated with menopause: Prevalence and quality of life burden. Menopause.

[28] Whiteley J., Wagner J.-S., Bushmakin A., Kopenhafer L., DiBonaventura M., Racketa J. (2013). Impact of the severity of vasomotor symptoms on health status, resource use, and productivity. Menopause.

[29] Saunders J.B., Aasland O.G., Babor T.F., De la Fuente J.R., Grant M. (1993). Development of the alcohol use disorders identification test (AUDIT): WHO collaborative project on early detection of persons with harmful alcohol consumption-II. Addiction.

[30] Kim K., Chang Y., Ahn J., Yang H.-J., Ryu S. (2020). Low levels of alcohol consumption and risk of intestinal metaplasia: A cohort study. Cancer Epidemiol. Prev. Biomark..

[31] Anderson D.J., Chung H.-F., Seib C.A., Dobson A.J., Kuh D., Brunner E.J., Crawford S.L., Avis N.E., Gold E.B., Greendale G.A. (2020). Obesity, smoking, and risk of vasomotor menopausal symptoms: A pooled analysis of eight cohort studies. Am. J. Obstet. Gynecol..

[32] Melby M.K., Anderson D., Sievert L.L., Obermeyer C.M. (2011). Methods used in cross-cultural comparisons of vasomotor symptoms and their determinants. Maturitas.

[33] Herber-Gast G.-C.M., Mishra G.D., van der Schouw Y.T., Brown W.J., Dobson A.J. (2013). Risk factors for night sweats and hot flushes in midlife: Results from a prospective cohort study. Menopause.

[34] Royston P., Parmar M.K. (2002). Flexible parametric proportional-hazards and proportional-odds models for censored survival data, with application to prognostic modelling and estimation of treatment effects. Stat. Med..

[35] Heeb J.-L., Gmel G. (2005). Measuring alcohol consumption: A comparison of graduated frequency, quantity frequency, and weekly recall diary methods in a general population survey. Addict. Behav..

[36] Hyde Riley E., Inui T.S., Kleinman K., Connelly M.T. (2004). Differential association of modifiable health behaviors with hot flashes in perimenopausal and postmenopausal women. J. Gen. Intern. Med..

[37] Herber-Gast G., Brown W.J., Mishra G.D. (2015). Hot flushes and night sweats are associated with coronary heart disease risk in midlife: A longitudinal study. BJOG Int. J. Obstet. Gynaecol..

[38] Gast G.C., Pop V.J., Samsioe G.N., Grobbee D.E., Nilsson P.M., Keyzer J.J., Wijnands-van Gent C.J., van der Schouw Y.T. (2011). Vasomotor menopausal symptoms are associated with increased risk of coronary heart disease. Menopause.

[39] Rinaldi S., Peeters P., Bezemer I., Dossus L., Biessy C., Sacerdote C., Berrino F., Panico S., Palli D., Tumino R. (2006). Relationship of alcohol intake and sex steroid concentrations in blood in pre-and post-menopausal women: The European Prospective Investigation into Cancer and Nutrition. Cancer Causes Control.

[40] Ginsburg E.S., Walsh B.W., Shea B.F., Gao X., Gleason R.E., Barbieri R.L. (1995). The effects of ethanol on the clearance of estradiol in postmenopausal women. Fertil. Steril..

[41] Randolph J.F., Sowers M., Bondarenko I., Gold E.B., Greendale G.A., Bromberger J.T., Brockwell S.E., Matthews K.A. (2005). The relationship of longitudinal change in reproductive hormones and vasomotor symptoms during the menopausal transition. J. Clin. Endocrinol. Metab..

[42] Schilling C., Gallicchio L., Miller S.R., Langenberg P., Zacur H., Flaws J.A. (2007). Genetic polymorphisms, hormone levels, and hot flashes in midlife women. Maturitas.

[43] Yoda T., Crawshaw L.I., Nakamura M., Saito K., Konishi A., Nagashima K., Uchida S., Kanosue K. (2005). Effects of alcohol on thermoregulation during mild heat exposure in humans. Alcohol.

[44] Kronenberg F., Downey J.A. (1987). Thermoregulatory physiology of menopausal hot flashes: A review. Can. J. Physiol. Pharmacol..

[45] Jones H., Bailey T.G., Barr D.A., France M., Lucas R.A., Crandall C.G., Low D.A. (2019). Is core temperature the trigger of a menopausal hot flush?. Menopause.

[46] Pachman D.R., Jones J.M., Loprinzi C.L. (2010). Management of menopause-associated vasomotor symptoms: Current treatment options, challenges and future directions. Int. J. Women’s Health.

[47] Berendsen H.H. (2000). The role of serotonin in hot flushes. Maturitas.

[48] Stockwell T., Zhao J., Sherk A., Rehm J., Shield K., Naimi T. (2018). Underestimation of alcohol consumption in cohort studies and implications for alcohol’s contribution to the global burden of disease. Addiction.

