# Supplementary material for: Alcohol Consumption Patterns and Risk of Early-Onset Vasomotor Symptoms in Premenopausal Women
Source: Nutrients. 2022 May 29;14(11):2276. doi: 10.3390/nu14112276 (PMC9182895; doi:10.3390/nu14112276)
Supplement: Supplementary file 1 [file nutrients-14-02276-s001.zip › nutrients-1734233-supplementary.pdf]

Supplemental Table S1. Cross-sectional and longitudinal association between alcohol consumption and risk of early-onset, moderately to severely bothersome VMS (overall and each components including hot flash or night sweat) among premenopausal women after further adjustment for hypertension and diabetes.

Alcohol drinking patterns	Multivariable-adjusted OR† (95% CI)			Multivariable-adjusted HR‡ (95% CI)		
	Vasomotor symptoms	Hot flush symptoms	Night sweat symptoms	Vasomotor symptoms	Hot flush symptoms	Night sweat symptoms
**Lifetime drinking status**						
Lifetime abstainer	Reference	Reference	Reference	Reference	Reference	Reference
Current drinker						
0.1 to <10 g/day	1.43 (1.02-2.00)	1.31 (0.92-1.87)	1.53 (0.88-2.68)	1.10 (0.85-1.41)	1.00 (0.75-1.35)	1.21 (0.89-1.64)
10 to <20 g/day	1.98 (1.26-3.10)	1.72 (1.07-2.77)	2.79 (1.40-5.59)	1.03 (0.70-1.51)	0.82 (0.50-1.33)	1.11 (0.70-1.76)
20 to <40 g/day	2.02 (1.17-3.50)	1.17 (0.61-2.25)	4.28 (2.03-9.04)	1.72 (1.06-2.79)	1.29 (0.71-2.37)	1.96 (1.13-3.42)
≥40 g/day	3.47 (1.69-7.09)	2.02 (0.88-4.68)	8.00(3.26-19.63)	2.22 (1.17-4.23)	1.32 (0.52-3.33)	3.14 (1.57-6.28)
P_{trend}	<0.01	0.06	<0.01	0.02	0.69	<0.01
Former drinker	1.06 (0.57-2.00)	1.06 (0.55-2.05)	1.65 (0.65-4.14)	1.27 (0.79-2.04)	1.06 (0.60-1.89)	1.15 (0.63-2.09)
**Frequency of drinking (days/week)**						
0	Reference	Reference	Reference	Reference	Reference	Reference
1-2	1.44 (1.05-1.96)	1.31 (0.95-1.82)	1.56 (0.95-2.58)	1.07 (0.85-1.37)	1.00 (0.75-1.32)	1.18 (0.89-1.57)
3	2.08 (1.35-3.19)	1.58 (0.99-2.54)	3.01 (1.61-5.65)	1.49 (1.03-2.13)	1.32 (0.85-2.04)	1.61 (1.06-2.46)
P_{trend}	<0.01	0.04	<0.01	0.06	0.37	0.04
**Number of drinks a drinking day**						
0	Reference	Reference	Reference	Reference	Reference	Reference
1-2	1.39 (1.01-1.93)	1.31 (0.93-1.84)	1.32 (0.79-2.22)	1.04 (0.81-1.33)	0.96 (0.72-1.28)	1.14 (0.85-1.54)
3-5	1.73 (1.21-2.48)	1.53 (1.05-2.23)	2.10 (1.21-3.65)	1.20 (0.90-1.59)	0.98 (0.70-1.39)	1.41 (1.00-1.97)
≥6	2.11 (1.34-3.34)	1.52 (0.91-2.54)	2.93 (1.51-5.68)	1.47 (1.00-2.17)	1.31 (0.82-2.09)	1.66 (1.05-2.62)

P_{trend}	<0.01	0.03	<0.01	0.03	0.44	<0.01

Abbreviations: BMI, body mass index; CI, confidence interval; OR, odds ratio; HR, hazard ratio; VMS, vasomotor symptoms.

†Logistic regression model was used to calcuate odds ratio and 95% confidence intervals for moderate-to-severe VMS.

‡ Parametric proportional hazard models were used to estimate hazard ratios (HRs) and 95% CIs for incident VMS.

The multivariable model was adjusted for age, attainment, smoking, physical activity level, BMI, hypertension, and diabetes in both cross-sectional and cohort studies.

Supplemental Table S2. Prevalence and incidence of early-onset, moderately to severely bothersome VMS among premenopausal women by frequency of binge drinking**.**

Prevalence of vasomotor symptoms	Vasomotor symptoms		Hot flush symptoms		Night sweat symptoms	
	Age-adjusted OR (95% CI)	Multivariable-adjusted OR (95% CI) *	Age-adjusted OR (95% CI)	Multivariable-adjusted OR (95% CI) *	Age-adjusted OR (95% CI)	Multivariable-adjusted OR (95% CI) *
**Frequency of binge drinking (*n* = 3322)** †						
Never	Reference	Reference	Reference	Reference	Reference	Reference
<once a month	1.36 (1.05-1.76)	1.30 (1.00-1.70)	1.27 (0.96-1.67)	1.19 (0.90-1.59)	1.76 (1.19-2.60)	1.71 (1.14-2.54)
once a month	1.72 (1.16-2.56)	1.71 (1.15-2.56)	1.53 (0.99-2.36)	1.47 (0.95-2.28)	2.30 (1.31-4.06)	2.32 (1.30-4.13)
weekly or more	1.60 (1.09-2.35)	1.45 (0.97-2.17)	1.28 (0.83-1.98)	1.14 (0.72-1.79)	2.71 (1.62-4.52)	2.42 (1.40-4.18)
P_{trend}	<0.01	<0.01	0.04	0.15	<0.01	<0.01
**Incidence of vasomotor symptoms**	**Vasomotor symptoms**		**Hot flush symptoms**		**Night sweat symptoms**	
	Age-adjusted HR (95% CI)	Multivariable-adjusted HR (95% CI) *	Age-adjusted HR (95% CI)	Multivariable-adjusted HR (95% CI) *	Age-adjusted HRs (95% CI)	Multivariable-adjusted HR (95% CI) *
**Frequency of binge drinking (*n* = 1906)** ‡						
Never	Reference	Reference	Reference	Reference	Reference	Reference
<once a month	1.00 (0.79-1.26)	0.98 (0.77-1.23)	0.97 (0.73-1.29)	0.94 (0.70-1.26)	1.01 (0.77-1.32)	0.99 (0.75-1.30)
once a month	1.42 (1.00-1.02)	1.35 (0.95-1.91)	1.31 (0.84-2.04)	1.27 (0.81-1.98)	1.50 (1.01-2.23)	1.39 (0.93-2.08)
weekly or more	1.57 (1.12-2.21)	1.54 (1.08-2.19)	1.45 (0.95-2.22)	1.45 (0.93-2.25)	1.67 (1.13-2.45)	1.58 (1.06-2.35)
P_{trend}	0.01	0.01	0.08	0.12	<0.01	0.02

Abbreviations: BMI, body mass index; CI, confidence interval; OR, odds ratio; HR, hazard ratio; VMS, vasomotor symptoms.

†Logistic regression models with robust variance were used to estimate odds ratios (ORs) and 95% CIs for prevalent VMS.

‡ Parametric proportional hazard models were used to estimate hazard ratios (HRs) and 95% CIs for incident VMS.

*Multivariable model was adjusted for age, education level, physical activity, BMI, and smoking status in both cross-sectional and cohort studies.

Supplemental Table S3. Cohort study characteristics of premenopausal women without vasomotor symptoms at baseline by drinking category (n=2,394).

Characteristics	Total	Drinking status					
		Lifetime abstainer	Former drinker	0 to <10 g/day	10 to <20 g/day	20 to <40 g/day	≥40 g/day
Number (%)	2,394 (100)	323 (13.5)	103 (4.3)	1,687 (70.5)	185 (7.7)	71 (3.0)	25 (1.0)
Age (years) *	44.6 ± 2.3	45.3 ± 2.5	44.9 ± 2.5	44.5 ± 2.3	44.5 ± 2.2	44.6 ± 2.4	44.4 ± 2.7
Age at menarche (years) *	13.9 ± 1.4	14.0 ± 1.4	13.8 ± 1.4	13.9 ± 1.4	13.9 ± 1.4	13.9 ± 1.3	14.0 ± 1.7
Obesity (%)[a]	15.6	16.1	25.2	14.9	16.2	15.5	12.0
High physical activity (%)[b]	14.9	11.8	13.6	14.5	18.9	29.6	16.0
Current smoker (%)	1.4	0.0	0.0	1.1	2.8	11.4	8.0
High education (%)[c]	81.8	82.0	82.4	83.0	76.4	69.6	72.0
Hypertension (%)[d]	3.2	5.3	1.0	2.7	3.2	8.5	8.0
Systolic BP (mmHg) *	103.2 ± 11.1	103.7 ± 11.4	103.5 ± 11.3	102.8 ± 11.0	103.7 ± 10.2	107.7 ± 13.6	108.3 ± 10.8
Diastolic BP (mmHg) *	66.3 ± 8.6	66.0 ± 8.5	64.9 ± 9.6	66.1 ± 8.4	67.2 ± 8.6	70.2 ± 10.8	72.1 ± 9.7
Diabetes (%)[e]	1.7	1.6	0.0	1.6	2.2	4.2	4.0
Glucose (mg/dL) *	92.6 ± 11.2	92.1 ± 10.0	91.5 ± 7.5	92.4 ± 11.3	94.2 ± 8.8	98.3 ± 20.0	93.3 ± 8.1
LDLC (mg/dL) *	118.3 ± 28.6	122.6 ± 28.6	120.3 ± 30.1	117.6 ± 27.9	118.8 ± 29.7	114.9 ± 34.3	103.5 ± 34.9
HDLC (mg/dL) *	67.5 ± 16.0	65.7 ± 15.7	65.4 ± 14.7	67.2 ± 15.8	69.7 ± 16.2	74.2 ± 18.2	82.5 ± 21.4
Triglycerides (mg/dL) †	73.0 (56.0-99.0)	74.0 (56.0-104.0)	75.0 (55.0-104.0)	71.0 (56.0-96.0)	81.0 (58.0-104.0)	80.0 (62.0-105.0)	77.0 (60.0-90.5)
AST (U/l) †	17.0 (15.0-20.0)	18.0 (15.0-20.0)	18.0 (16.0-20.0)	17.0 (15.0-20.0)	17.0 (15.0-20.0)	19.0 (16.0-21.0)	18.0 (16.0-19.0)
ALT (U/l) †	13.0 (11.0-17.0)	14.0 (11.0-17.0)	14.0 (11.0-18.0)	13.0 (11.0-17.0)	13.0 (11.0-17.0)	13.0 (12.0-17.0)	14.0 (11.0-16.0)
GGT (U/l) †	13.0 (10.0-18.0)	13.0 (10.0-17.0)	14.0 (11.0-17.0)	13.0 (10.0-17.0)	15.0 (12.0-21.0)	15.0 (11.0-20.0)	18.0 (15.0-31.0)
HOMA-IR†	1.1 (0.7-1.6)	1.0 (0.7-1.5)	1.1 (0.8-1.9)	1.1 (0.7-1.6)	1.2 (0.8-1.6)	1.1 (0.8-1.4)	0.8 (0.5-1.0)
hsCRP (mg/L)†	0.03 (0.02-0.06)	0.03 (0.02-0.06)	0.03 (0.02-0.05)	0.03 (0.02-0.06)	0.03 (0.02-0.06)	0.04 (0.02-0.08)	0.03 (0.02-0.06)

Abbreviations: MET, metabolic equivalents; AST, aspartate aminotransferase; ALT, alanine aminotransferase; BP, blood pressure; GGT, gamma-glutamyl transpeptidase; HDL-C, high-density lipoprotein cholesterol; HOMA-IR, homeostasis model assessment of insulin resistance; hsCRP, high-sensitivity C-reactive protein; LDLC, low-density lipoprotein cholesterol

Data presented as *mean ± standard deviation, †median (interquartile range), or percentage.

[a] body mass index ≥ 25kg/m2; [b] defined as either > 3 days of vigorous activities achieving at least 1500 MET min/week, or > 7 days of any combination of walking and moderate or vigorous activities achieving at least 3000 MET min/week; [c] ≥college graduate; [d] defined as either blood pressure ≥140/90 mmHg, history of physician-diagnosed hypertension, or antihypertensive medication use; [e] glucose-lowering medication use, fasting hyperglycemia based on either serum glucose of ≥126 mg/dL, or glycated hemoglobin of ≥6.5%

Supplemental Table S4. Longitudinal association between alcohol consumption and incidence of early-onset bothersome vasomotor symptoms among premenopausal women by alcohol flushing.

Alcohol drinking patterns	Person-Years	Incident cases	Incidence rate (Cases per 100 PY)	Age-adjusted HRs (95% CI)	Multivariable-adjusted HR (95% CI) *
**Alcohol non-flushers (n = 1631)**					
Lifetime abstainer	642.1	28	4.4	Reference	Reference
Current drinker					
0.1 to <10 g/day	5517.5	257	4.7	1.19 (0.81—1.76)	1.24 (0.84—1.84)
10 to <20 g/day	760.0	38	5.0	1.30 (0.80—2.12)	1.21 (0.74—1.98)
20 to <40 g/day	287.1	20	7.0	1.86 (1.05—3.31)	1.82 (1.01—3.28)
≥40 g/day	104.1	9	8.6	2.52 (1.18—5.37)	2.38 (1.11—5.11)
P_{trend}				<0.01	0.02
Former drinker	246.1	14	5.6	1.48 (0.78—2.82)	1.50 (0.79—2.87)
**Alcohol flushers (n = 713)**					
Lifetime abstainer	752.2	35	4.7	Reference	Reference
Current drinker					
0.1 to <10 g/day	2338.4	115	4.9	1.24 (0.84—1.85)	1.21 (0.82—1.79)
10 to <20 g/day	82.8	2	2.4	0.66 (0.16—2.77)	0.71 (0.17—2.96)
20 to <40 g/day	24.6	2	8.1	2.59 (0.61—10.89)	2.40 (0.54—10.71)
≥40 g/day	16.8	2	11.9	2.06 (0.49—8.67)	2.44 (0.55—10.93)
P_{trend}				0.20	0.21
Former drinker	137.1	8	5.8	1.44 (0.66—3.11)	1.24 (0.56—2.71)

Abbreviations: CI, confidence interval; HR, hazard ratio; PY, person-years; VMS, vasomotor symptoms

P=0.87 for interaction between alcohol consumption and alcohol flushing response for moderate-to-severe VMS.

* Estimated from parametric proportional hazard models. The multivariable model was adjusted for age, attainment, smoking, physical activity level, BMI, hypertension, and diabetes
